# TGFβ2-induced senescence during early inner ear development

**DOI:** 10.1038/s41598-019-42040-0

**Published:** 2019-04-11

**Authors:** Alejandro Gibaja, María R. Aburto, Sara Pulido, Manuel Collado, Juan M. Hurle, Isabel Varela-Nieto, Marta Magariños

**Affiliations:** 1 Instituto de Investigaciones Biomédicas “Alberto Sols” (CSIC-UAM), Madrid, Spain; 2 CIBERER, Instituto de Salud Carlos III, Madrid, Spain; 3 Instituto de Investigación Sanitaria de Santiago de Compostela, Santiago de Compostela, Spain; 4 Departamento de Anatomía y Biología Celular and IDIVAL, Universidad de Cantabria, Santander, Spain; 5 Instituto de Investigación Sanitaria del Hospital Universitario La Paz (IdiPAZ), Madrid, Spain; 6 Departamento de Biología, Universidad Autónoma de Madrid, Madrid, Spain

## Abstract

Embryonic development requires the coordinated regulation of apoptosis, survival, autophagy, proliferation and differentiation programs. Senescence has recently joined the cellular processes required to master development, in addition to its well-described roles in cancer and ageing. Here, we show that senescent cells are present in a highly regulated temporal pattern in the developing vertebrate inner ear, first, surrounding the otic pore and, later, in the otocyst at the endolymphatic duct. Cellular senescence is associated with areas of increased apoptosis and reduced proliferation consistent with the induction of the process when the endolymphatic duct is being formed. Modulation of senescence disrupts otic vesicle morphology. Transforming growth factor beta (TGFβ) signaling interacts with signaling pathways elicited by insulin-like growth factor type 1 (IGF-1) to jointly coordinate cellular dynamics required for morphogenesis and differentiation. Taken together, these results show that senescence is a natural occurring process essential for early inner ear development.

## Introduction

The vertebrate inner ear is the organ that senses sound, movement and position. Most of the cell types that populate the adult vestibular and auditory parts of the inner ear have a common developmental origin in the otic placode^1^. These cell types include sensory hair cells, supporting cells, secretory cells and neurons for the vestibular and acoustic ganglia, all spatially organized with a fine-tuned geometry matched for the functions of hearing and equilibrium^2^. The developmental processes that give rise to such precise structure involve cell differentiation and morphogenetic events. Once otic identity is established in the neuroectoderm, the otic placode invaginates to form the otic cup, which closes up giving rise to the otic vesicle. The acoustic-vestibular ganglion neurons specify at the ventral part of the otic cup and vesicle, neuroblasts delaminate and migrate towards the neural tube^3^. In parallel, the otic vesicle cells undergo proliferation, apoptosis and differentiation. Cellular dynamics underlie morphogenetic movements that generate the primordium of the different sensory receptors, as well as the endolymphatic duct and other structures of the adult inner ear. Apoptosis is crucial for the closure of the otic pore, shaping the otic vesicle, the migration of the epithelial neuroblasts and for adjusting the numbers of adult neurons^4,5^. The efficient removal of these damaged or unnecessary cells requires energy that is provided by autophagy^6^.

Factors secreted by otic and neighbouring cells participate in regional morphogenesis establishing the dorsoventral axis of the otic vesicle. WNT and bone morphogenetic protein (BMP) signalling are involved in the determination of the dorsal vestibular region responsible for balance perception, whereas sonic hedgehog (SHH) signalling is required for specification of the ventral auditory region^7^.

Cell senescence is defined as a stable cell-cycle arrest elicited in response to damage, such as intense oncogenic signalling, DNA damage and telomere loss^8^. Senescent cells express inhibitors of cyclin dependent kinases, as *p*2*1*, *p15* and *p16*, whereas the expression of cell cycle-promoting genes is suppressed. Senescent cells also show increased expression of the lysosomal β-galactosidase enzyme, a feature manifested as stronger senescence-associated β-galactosidase staining (SAβG), and present a secretory profile known as the senescence-associated secretory phenotype (SASP) that include soluble factors that modulate the cellular microenvironment^9^. Senescence has been classically considered a tumour suppressor mechanism and also a sign of aging^10,11^. More recently it has been shown that cellular senescence is a developmental program involved in the remodelling or elimination of the endolymphatic sac, the mesonephros, the neural tube, the apical ectodermal ridge (AER) and the interdigital tissue^12–14^. Developmental senescence acts together with apoptosis in the elimination of unwanted cells as well as in patterning and morphogenesis^14^. It shares most of the cellular characteristics of oncogene-induced senescence, such as arrested proliferation, increased SAβG staining and a secretory phenotype. But not all the mediators, only p21 plays an important role in mammalian developmental senescence^12,13^. In the chicken embryo, the tumour suppressor genes *Btg1* and *Btg2* are associated with developmental senescence^14^.

Extrinsic factors and intracellular signalling regulate cellular senescence in a tissue-specific manner. In the mouse endolymphatic duct, the activation of the phosphoinositide 3-kinase (PI3K)/FOXO pathway is a negative regulator of developmental cell senescence, whereas TGFβ/SMAD is an inductor^12^. In the mouse AER, the RAF-MEK-ERK pathway instructs senescence in neighbouring mesenchymal cells^13^. Interestingly, senescent cells secrete TGFβ, IGFBPs and cytokines among other pro-inflammatory, anti-proliferative and pro-apoptotic signals^15^.

Insulin-like growth factor 1 (IGF-1) belongs to the insulin family of peptides and it is a potent pro-survival factor for epithelial and neuronal otic populations via the activation of the PI3K/AKT pathway^16,17^. IGF-1 also activates the RAF-MEK-ERK cascade to promote the proliferation of otic progenitors^18,19^. IGF-1 and its receptor IGF1R are expressed during vertebrate otic development^20^, where IGF-1 is essential for the differentiation and survival of mouse auditory neurons^21^. Moreover, IGF-1 deficiency is a rare human disease associated with syndromic deafness^22^. On the other hand, TGFβ superfamily includes BMPs, TGFβs, activins and nodal, which bind to type I and II receptors (TGFRI and TGFRII) forming the active tetrameric receptors^23^. Cytoplasmic proteins are then recruited and phosphorylated by the active receptor. Typically receptor-activated SMAD proteins (R-SMAD) form heteromeric complexes with the common mediator SMADs (Co-SMAD). R-SMAD/Co-SMAD complexes translocate to the nucleus and regulate the expression of target genes. BMPs and TGFβ bind different TGFβ receptors and activate different SMAD proteins^24^, showing distinct downstream signalling traits. Further modulation is exerted by additional cell surface receptors like TGFβR3 that also modulates the cellular response to TGFβ2^23^, as well as transmembrane proteins, like endoglin, that associate with receptors to further modulate their activity^25^. The role of BMP ligands has been extensively studied in the otic vesicle^26,27^, however, the role of TGFβ2 has not been investigated to date.

Here, we show the presence of cellular senescence during early otic development of the chicken embryo. Positive senescent cells in the developing inner ear show temporal and regional-specific patterns. Cells of the endolymphatic duct show intense senescence associated with apoptosis and reduced proliferation. We also observed that both navitoclax elimination of senescent otic cells and palbociclib induction of senescence disrupt the morphology of the otocyst. Distinct expression patterns of TGFβ factors and receptors as well as regulation of downstream mediators supported the key role of the TGFβ superfamily during otic early development. Thus, TGFβ2 promotes senescence in the developing inner ear, whilst its inhibition abolishes SMAD2 phosphorylation and reduces TGFβ2-induced SAβG. Further studies on coregulatory mechanisms showed the participation of RAF-MEK-ERK and the PI3K/AKT pathways in the modulation of otic developmental senescence.

## Material and Methods

### Chicken and mouse embryos

Chicken embryos were obtained from fertilized eggs from a local farm (Granja Santa Isabel, Cordoba, Spain) and they were incubated in a humidified atmosphere at 37.8 °C. Embryos were staged according to Hamburger and Hamilton’s (HH) criteria^28^. Mouse embryos were obtained from C57BL/6J mice that were purchased from Charles River (Barcelona, Spain) and housed under standard conditions. All experiments were approved by the CSIC Bioethics Committee and carried out in full accordance with the guidelines of the European Community (2010/63/EU) and the Spanish regulations (RD 53/2013).

### Embryo and tissue preparation for *in situ* hybridization and immunofluorescence

Whole embryos or tissues were dissected in phosphate-buffered saline (PBS) and fixed overnight in 4% (w/v) paraformaldehyde (PFA; Merck, Darmstadt, Germany) in PBS at 4 °C. Subsequently, embryos were cryoprotected overnight in 15% sucrose/PBS at 4 °C and then embedded at 37 °C in 15% sucrose/10% gelatine in PBS. Gelatine-embedded tissues were frozen in isopentane at −80 °C and sectioned (20 μm) at −25 °C in a cryostat (Cryocut 1900; Leica Microsystems, Deerfield, IL). The sections obtained were used for *in situ* hybridization or immunofluorescent staining as described^6,17,19^.

### Isolation, organotypic culture and treatment of otic vesicles

Embryos at stage HH18 (65 h of incubation) were obtained and the otic vesicles were dissected from the surrounding mesenchymal tissue with sharpened tungsten needles, they were transferred into four-well culture-plates (Nunc Roskilde, Denmark) and then incubated at 37 °C in a water-saturated atmosphere containing 5% CO_2_. The standard culture medium was M199 medium with Earle’s salts (Sigma Aldrich Química, Madrid, Spain) supplemented with 2 mM glutamine (Gibco, Paisley, UK) and antibiotics [50 IU/ml penicillin (Ern, Barcelona, Spain) and 50 mg/ml streptomycin (CEPA, Madrid, Spain)]. For immunostaining and TUNEL labelling otic vesicles were fixed for 2 h in PFA at 4 °C. For SAβG staining the otic vesicles were fixed for 10 min with the fixative solution provided by the Senescence β-Galactosidase Staining Kit (Cell Signaling Technology, Danvers, Massachusetts, USA).

*Ex vivo* cultures were performed as previously reported^6,16,17,19^. Explants were treated with either the BCL-2/BCL-XL-inhibitor navitoclax (1 µM, ABT263, Selleckchem, Houston, TX, USA), the CDK4/CDK6 inhibitor palbociclib (1 µM, PD0332991, Selleckchem), BOC (200 µM, Boc-D-fluoromethyl ketone, Calbiochem, La Jolla, CA, USA), TGFβ2 (10 ng/ml, PeproTech, Rocky Hill, NJ, USA), the TGFβR1 inhibitor SB431542 (10 µM, Sigma), IGF-1 (10 nM, recombinant IGF-1, Roche Molecular Biochemicals, Basel, Switzerland), sorafenib (2.5 µM, BAY 43–9006, Bayer HealthCare Pharmaceuticals, West Haven, CT, USA), or AKTi (50 µM, AKT inhibitor VIII, Calbiochem). The concentrations used did not show toxicity nor did the 0.01% dimethyl sulfoxide (DMSO) solvent. Otic vesicle explants cultured in serum-free medium without additives were used as experimental controls (0S).

Endolymphatic duct and dorsal areas were measured from light microscopy images using the FIJI package image processing based on ImageJ software (Wayne Rasband, National Institutes of Health, Bethesda, MD). Results are presented as the mean ± SEM of the percentage of the area of interest per total otic vesicle area. Values were normalized to the 0S explants.

### Quantitative RT-PCR

Otic vesicles were dissected out from chicken embryos and pooled to obtain RNA at different developmental stages: closure of the otic cup (HH17, n = 45–50), formation and growth of the otic vesicle (HH18, n = 30–40), endolymphatic duct emergence (HH19, n = 25–30) and a more differentiated stage (HH23, n = 5–10). Microdissection of HH19 otic explants was performed to isolate dorsal and ventral regions. Three to four independent pools of RNA from each stage were isolated with TRIZOL (Invitrogen, Carlsbad, CA) following the manufacturer’s instructions. The integrity and concentration of the RNA was assessed with an Agilent Bioanalyzer 2100 (Agilent Technologies) and cDNA was generated by reverse transcription, RT, (High Capacity cDNA Reverse Transcription Kit; Applied Biosystems). Quantitative PCR (qPCR) of each pool was performed in triplicate using specific oligonucleotides for the indicated genes. Specific primers for chicken genes would be provided upon request. Eukaryotic 18S rRNA and HPRT1 were used as endogenous housekeeping genes. SYBR Green qPCR was performed on a 7900HT Real-Time PCR System and gene expression was estimated as 2^−ΔΔCt^.

### Immunohistochemistry and immunofluorescence

Antibodies used for immunofluorescence are shown in Supplementary Information Table S1. Procedures used have been described elsewhere^6,17,19^. Levels of PH3 immunostaining and otic vesicle areas were quantified using FIJI software, in compiled confocal microscopy projections as reported^17,19^. Colour channels of the signals of interest were converted into grey scale images. Subsequently both the area and the intensity of the signal were measured, and normalized to the 0S condition, which was given an arbitrary value of 100. 3–6 samples per condition were assayed from 2–6 independent experiments.

### Western blotting

Otic vesicles (HH18, n = 25) were isolated and cultured for 20 h for protein extraction and analysis as reported^17,19^. Membranes were incubated overnight at 4 °C, with the primary antibodies indicated in Supplementary Information Table S1. Images of the blots were captured using an ImageQuant LAS4000 mini digital camera (GE Healthcare Bio-Sciences), and densities of the immunoreactive bands were quantified by densitometry using ImageQuant TL software (GE Healthcare Bio-Sciences).

### Apoptosis

Apoptotic cell death patterns were studied by Tdt-mediated dUTP nick-end labelling (TUNEL) of fragmented DNA using the kit Dead-EndTM Fluorometric TUNEL System (Promega, Madison, WI) as described by the manufacturer in frozen sections as reported^5^, or adapted to whole organ labelling^6,17,19^. Otic vesicles were mounted in Prolong Gold/DAPI and visualized by confocal microscopy. TUNEL-positive nuclei were quantified from compiled confocal microscopy projections by FIJI software and the results are presented as the mean ± SEM of the positive cells per total area. Values were normalized to those of the 0S explants.

### Senescence-associated beta-galactosidase staining

SAβG was performed by using the Senescence β-Galactosidase Staining Kit (Cell Signaling Technology). After culture and fixation, otic vesicles were washed in PBS and incubated with the X-Gal solution at pH 6 at 37 °C protected from light. For SAβG in whole embryos at stages HH17, HH18, HH19 and HH20, the embryos were fixed for 15 min and incubated in X-Gal solution. To perform the SAβG in chicken embryo tissue sections, the embryos were obtained, fixed for 15 min, frozen and sectioned. Sections were post-fixed for 2 min and incubated with the X-Gal solution.

### EdU labelling and SAβG staining of otic vesicles

Otic vesicles (HH18) were isolated and cultured for 20 h. EdU (5-ethynyl-2′-deoxyuridine, Invitrogen) was diluted in PBS-DMSO 1% and added for the last hour of the culture at a final concentration of 100 µM. Otic vesicles were fixed for 20 min and incubated with the X-Gal solution pH 6 at 37 °C. Then, otic vesicles were fixed with PFA, permeabilized with PBS-T, washed with 2% PBS-BSA and incubated with the Click-iT Reaction Cocktail (Invitrogen) with Alexa-488 or Alexa Fluor 594 (5 µM, Invitrogen) at RT in darkness for 30 min. Finally, they were washed again with 2% PBS-BSA and mounted in Vectashield with DAPI (Vector, Peterborough, UK).

SAβG staining levels and EdU labelling were quantified using the FIJI image processing software based on ImageJ. The colour channels of the signals of interest were converted into grey scale images. The blue staining area was analysed relative to the otic vesicle total area. Levels of EdU signal from compiled confocal microscopy were measured and relativized to otic vesicle total area. Data were normalized to the 0S condition, which was given an arbitrary value of 100.

### *In situ* hybridization

*In situ* hybridization with digoxigenin-labelled antisense RNA probes (1 mg/ml) was performed essentially as described previously with minor modifications^17^. Three HH18, HH20 and HH25 embryos were tested for *Tgfβ2* and three HH18 embryos were tested for *Tgfβr*2 expression in at least two independent experiments. Specificity of the signal was assessed by hybridization of a sense riboprobe^29^.

### Statistics

Results are shown as mean ± SEM and the statistical significance was determined with the Student’s t-test. A p < 0.05 was considered significant. *p < 0.05; **p < 0.01; ***p < 0.001.

### Ethical standards

The experiments comply with the current laws of the countries in which they were performed.

## Results

### Cellular senescence during early development of the inner ear

**Figure 1:**
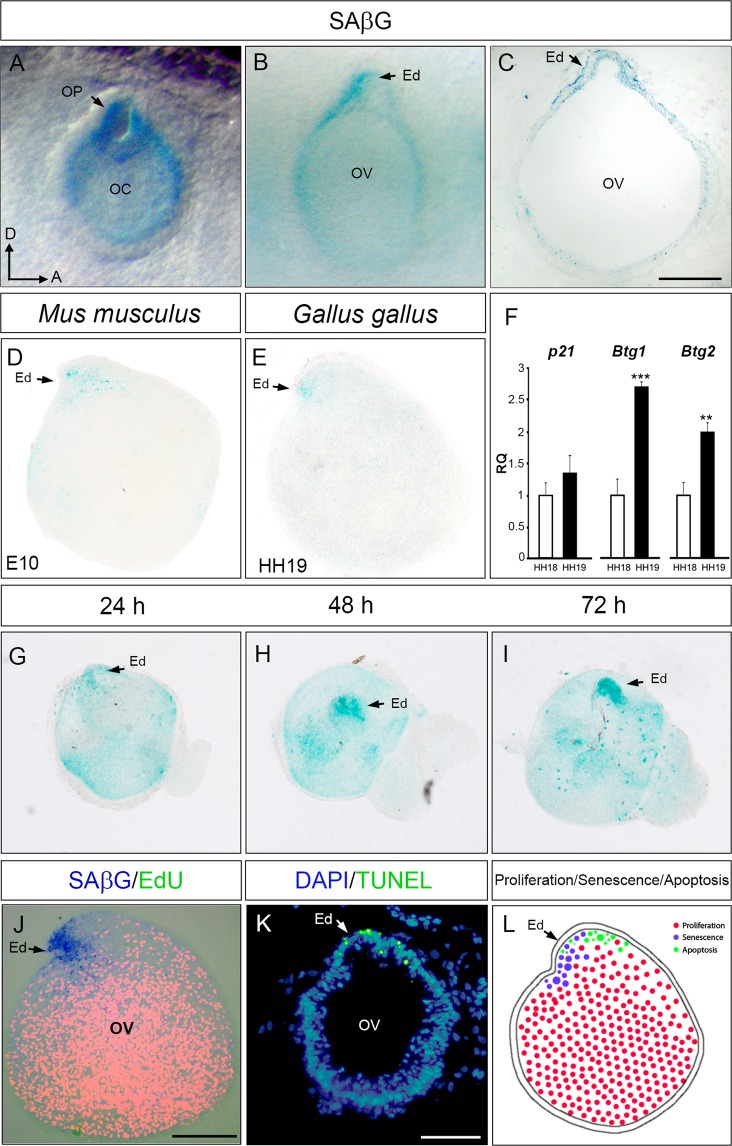
Senescence in the developing chicken inner ear. SAβG staining in the otic epithelium of chicken embryos at HH17 (**A**) and at HH20 (**B**) stages showing intense staining in the otic pore and in the developing endolymphatic duct (arrows). (**C**) Representative microphotograph of an otic vesicle cryosection SAβG stained. (**D**–**E**) SAβG staining in isolated otic vesicles from E10 mouse (**D**) and HH18 chicken (**E**) embryos showing specific labelling at the endolymphatic duct. (**F**) *p21*, *Btg1* and *Btg2* mRNA expression levels were measured by RT-qPCR in HH18 (white bars) and HH19 (black bars) otic vesicle explants. Gene expression was calculated as 2^−ΔΔCt^ and normalized to the levels at HH18. The results are expressed as the means ± SEM from three independent experiments performed in triplicate. Statistical significance was determined with the Student’s t-test: **P < 0.01, ***P < 0.005, versus HH18. (**G**–**I**) SAβG staining associated to the endolymphatic duct throughout early morphological changes in an otocyst *ex vivo* culture. Otic vesicles were isolated from HH18 chicken embryos and cultured for 24, 48 and 72 h in free-serum medium (0S). (**J**) Proliferation was evaluated by EdU incorporation (red) at HH18 otic vesicles cultured *ex vivo* for 20 h. (**K**) TUNEL labelling (green) in embryo sections showed the presence of apoptotic cells in the developing endolymphatic duct. (**L**) Drawing summarizing the localization of senescent, apoptotic and proliferative cells in the otic vesicle. Abbreviations: Ed, endolymphatic duct; OC, otic cup; OP, otic pore; OV, otic vesicle. Orientation: A, anterior; D, dorsal. Scale bar: 80 μm. Representative microphotographs are shown from at least n = 5 otic vesicles per condition.

β-galactosidase staining (SAβG) was used to study the presence of senescent cells in the developing inner ear in whole-mount HH17 to HH20 embryos (Supplementary Information Fig. S1). HH17 embryos showed marked staining of SAβG in the otic epithelium, with the highest levels observed around the otic pore (Fig. 1A), an area of known intense apoptosis^30^. Later in development (HH19-HH20), SAβG staining was intense in the endolymphatic duct (Fig. 1B,C). Dissected otic vesicles from mouse E10 and HH19 chicken embryos showed that senescence is highly restricted to the developing endolymphatic duct in both species (Fig. 1D,E), suggesting that otic senescence is dynamic and developmentally-regulated. To assess the time-window of otic senescence induction, expression levels of *p21*, *Btg1 and Btg2* were analysed by RT-qPCR in HH18 (white bars) and HH19 (black bars) otic vesicle explants. While *p21* expression was not significantly increased, *Btg1* and *Btg2* showed a significant increase (2.7-fold and 2-fold respectively) in HH19 compared to HH18 (Fig. 1F).

Isolated HH18 otic vesicles were cultured for 24, 48 and 72 h and the intense SAβG staining at the endolymphatic duct showed that senescence could be studied *ex vivo* (Fig. 1G–I, arrows). Endolymphatic duct SAβG-labelled senescent cells did not show EdU incorporation (Fig. 1J, arrow), thus, increased senescence was associated with reduced proliferation. Finally, TUNEL assay confirmed the presence of increased apoptosis in the endolymphatic duct (Fig. 1K, arrow). Figure 1L schematizes otocysts patterns of the above described senescent, apoptotic and proliferative cells. Taken together, these data suggest that developmental senescence contributes to sculpt the otocyst.

### Otic morphological changes are associated with modulation of senescence

**Figure 2:**
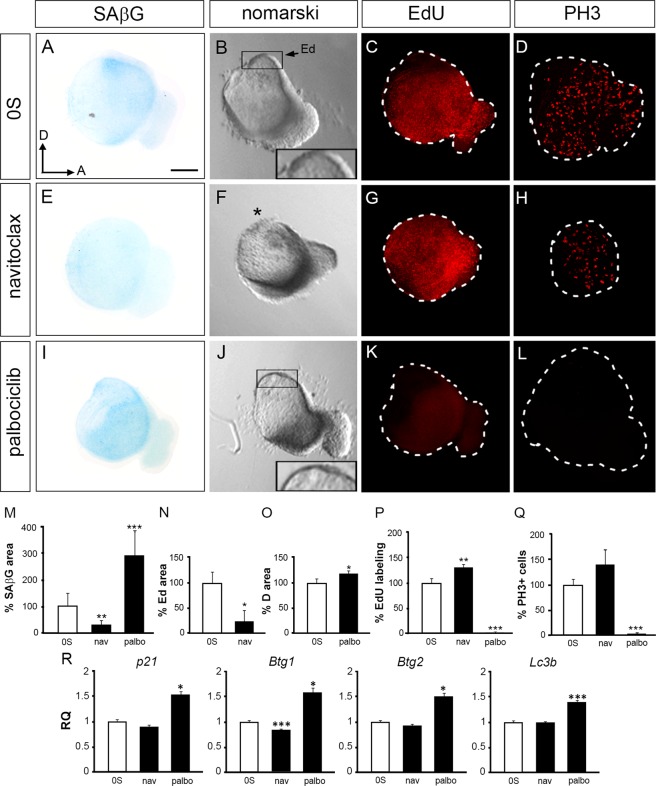
Senescence modulation disrupts early inner ear development. Otic vesicles were isolated from HH18 chicken embryos and cultured *ex vivo* for 20 h in serum free medium (0S) (**A**–**D**), in the presence of navitoclax (1 µM, **E**–**H**) or palbociclib (1 µM, **I**–**L**). SAβG staining in the otic epithelium of chicken embryos reveals the modulation of senescence levels (**A**,**E**,**I**), quantification in (**M**) data are represented as SAβG mean area ± SEM with respect to the otic vesicle area, normalized to the 0S condition. The statistical significance was determined by Student’s t-test: **P < 0.01, ***P < 0.005 versus control condition (0S, white bars). Light microscopy images are shown to visualise morphology (**B**,**F**,**J**). Navitoclax-treated otic vesicles showed reduction of the endolymphatic duct area (**F**, asterisk, quantification in **N**); note the expanded and thinned otocyst dorsal region caused by the palbociclib treatment in comparison with the control condition (**B**,**J**, insets; quantifications in **O**). Proliferation was measured by EdU incorporation (red; **C**,**G**,**K**, quantification in **P**) and by immunostaining for the mitotic marker phospho-Histone-3, PH3 (red; **D**,**H**,**L**, quantification in **Q**). Representative images from optical or compiled confocal microscopy are shown from at least six otic vesicles per condition studied, in at least three independent experiments. Quantification for areas and inmunostainings were measured as described in Materials and Methods. Data are shown as the mean ± SEM and the statistical significance was determined by Student’s t-test: *P < 0.05, **P < 0.01, ***P < 0.005 versus control condition (0S, white bars). Scale bar, 150 µm. (**R**) *p21*, *Btg1*, *Btg2* and *Lc3b* mRNA expression levels were measured by RT-qPCR in isolated HH18 otic vesicles cultured for 20 h. Gene expression was calculated as 2^−ΔΔCt^ and normalized to the levels of the control condition (0S, white bars). The results are expressed as the mean ± SEM from three independent experiments performed in triplicate. Statistical significance was determined with the Student’s t-test: *P < 0.05, ***P < 0.005, versus 0S. Ed, endolymphatic duct; Orientation: A, anterior; D, dorsal.

Otic vesicles were next incubated with navitoclax that specifically promotes apoptosis of senescent cells^31^. Navitoclax-treated otic vesicles showed a 70% reduction of SAβG staining (Fig. 2A,E, quantification in M), a rounded morphology and a reduction in the endolymphatic duct area (0.8-fold, Fig. 2B,F, asterisk, quantification in N). EdU incorporation and immunofluorescence for mitotic cells with PH3 showed an increment of cells in the S-phase in navitoclax-treated otic vesicles (1.3-fold, Fig. 2C,D,G,H, quantification in P-Q). The elimination of senescent cells with navitoclax also increased the number of apoptotic cells (Supplementary Information Fig. S2). Conversely, reduction of apoptosis by treatment with the pan-caspase inhibitor BOC increased SAβG staining (Supplementary Information Fig. S2). These results suggest a link between apoptosis and senescence during endolymphatic duct formation.

To promote senescence, explants were treated with palbociclib^32^. After 20 h in culture, palbociclib treatment promoted a 2.9-fold increment of SAβG staining (Fig. 2A,I, quantification in M) in otic vesicles showing an expanded and thinned dorsal region (Fig. 2B,J, insets, quantification in O). Induction of senescence with palbociclib was associated with the absence of proliferation markers (Fig. 2C,D,K,L, quantification in P-Q). To further document navitoclax and palbociclib actions, we examined gene expression levels of *p21*, *Btg1 and Btg2*, which were upregulated by palbociclib (1.52-, 1.57- and 1.48-fold respectively; Fig. 2R). Finally, considering that it has been reported that the highly secretory senescent cells require induction of autophagy to restore cell homeostasis^33^, we measured *Lc3b* (*microtubule-associated protein light chain 3 beta*, widely used as an autophagy biomarker) transcription levels that indeed were increased in otic vesicles incubated with palbociclib (1.38-fold; Fig. 2R).

In summary, we show that otic vesicle morphogenesis can be modulated by drugs that increase or reduce cellular senescence, which particularly impact the formation of the endolymphatic duct.

### TGFβ family factors and their receptors are expressed in the developing inner ear

Senescent cells have a secretory phenotype that is essential to modify their cellular microenvironment, with TGFβ family members featuring among the secreted factors^15^. To explore the mediator that induces senescence in the endolymphatic duct, we considered the actions of TGFβ/BMP signalling with a special interest in TGFβ2, a factor that has not been explored in this context before.

**Figure 3:**
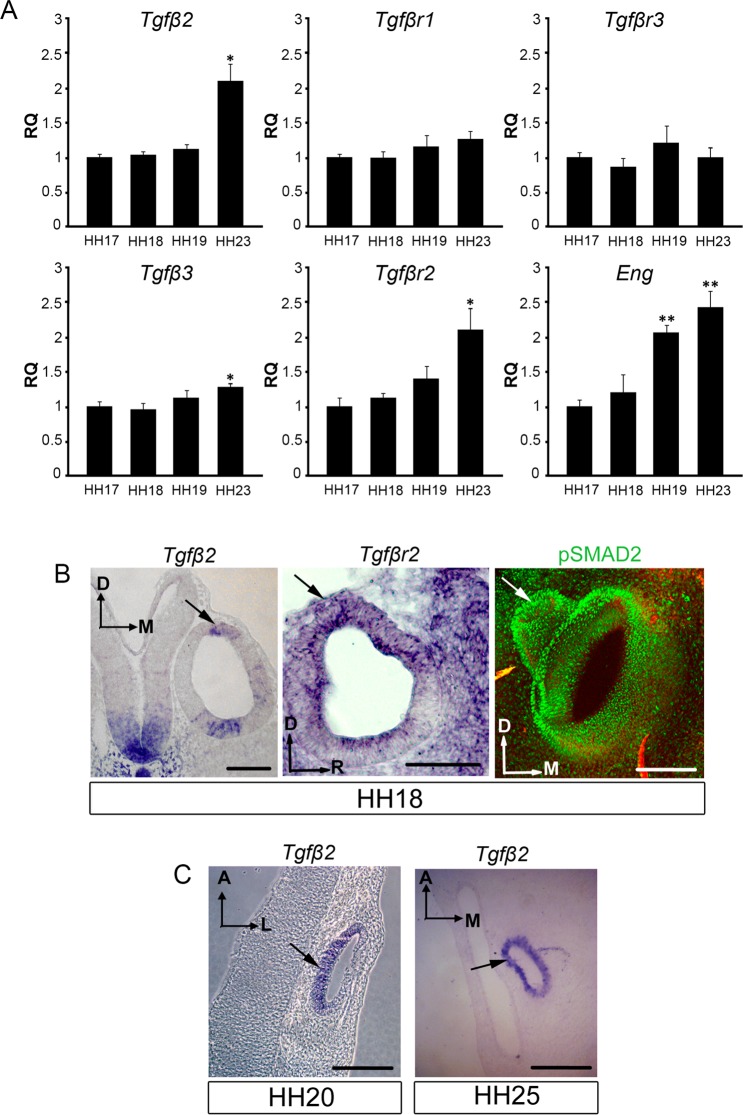
TGFβ pathway expression in the inner ear during early development. (**A**) Expression of *Tgfβ2*, *Tgfβ3*, *Tgfβr1*, *Tgfβr2*, *Tgfβr3* and *Eng* mRNA in the inner ear was measured by RT-qPCR at the indicated developmental stages. Eukaryotic 18s rRNA was used as an endogenous control gene. Gene expression was calculated as 2^−ΔΔCt^ and normalized to the HH17 levels. The results are expressed as mean ± SEM from four independent experiments. (**B**) TGFβ pathway components (*Tgfβ2*, *Tgfβr2* and pSMAD2) expression in inner ear sections. At HH18 *Tgfβ2* was expressed in the dorsal area of the otic epithelium, where the endolymphatic duct will emerge (first panel, arrow). *Tgfβr2* was expressed predominantly in the dorsal area of the otic epithelium, including the endolymphatic duct anlagen (second panel, arrow). The presence of active TGFβ signalling was assessed with immunodetection of the phosphorylated downstream effector protein SMAD2 (pSMAD2), which showed a nuclear localization throughout the otic epithelium and the developing endolymphatic duct (third panel, arrow). (**C**) *Tgfβ2* was strongly expressed in the developing endolymphatic duct at later developmental stages, HH20 and HH25 (arrows). Representative microphotographs are shown from at least three embryos per condition. Orientation: A, anterior; D, dorsal; L, lateral; M, medial; R, rostral. Scale bars: 100 μm.

The expression of *Tgfβ2*, *Tgfβ3*, *Tgfβr1*, *Tgfβr2*, *Tgfβr3 and Eng* was studied by RT-qPCR in inner ear extracts from HH17-HH19 and HH23 embryonic stages (Fig. 3A). All the analysed transcripts were expressed in the studied stages, but with distinct temporal patterns. *Tgfβr1* and *Tgfβr3* expression levels did not vary, but, in contrast, *Tgfβ2*, *Tgfβ3* and the receptor *Tgfβr2* transcripts showed a significant increase at HH23, whereas the co-receptor *Eng* increased its expression at stage HH19 that persisted to HH23. These data were further confirmed as the expression of TFGβ-system genes in HH23 inner ear explants was associated with areas of increased SAβG staining (data not shown).

The cellular expression pattern of the transcript for *Tgfβ2* was further studied by *in situ* hybridization (Fig. 3B,C). *Tgfβ2* mRNA presented a localized expression at stage HH18 in the endolymphatic duct precursor area (Fig. 3B first panel, arrow). Consistent with TGFβ2 signalling, we found typical nuclear immunopositivity for the phosphorylated SMAD2 effector protein (pSMAD2) in the otic epithelium of the otic vesicle and in the endolymphatic duct anlagen (Fig. 3B, third panel). *Tgfβr2* was found to be strongly expressed in the dorsal part of the otic epithelium, which includes the endolymphatic duct anlagen (Fig. 3B, second panel, arrow). We also investigated the presence of *Tgfβ2* at later developmental stages and found strong expression in the developing endolymphatic duct at stages HH20 and HH25 (Fig. 3C, arrows).

Thus, our results show that the TGFβ signalling is active in the otic epithelium during early inner ear development, with specific expression of TGFβ2 in the endolymphatic duct area.

### TGFβ2 promotes senescence in the otic vesicle

**Figure 4:**
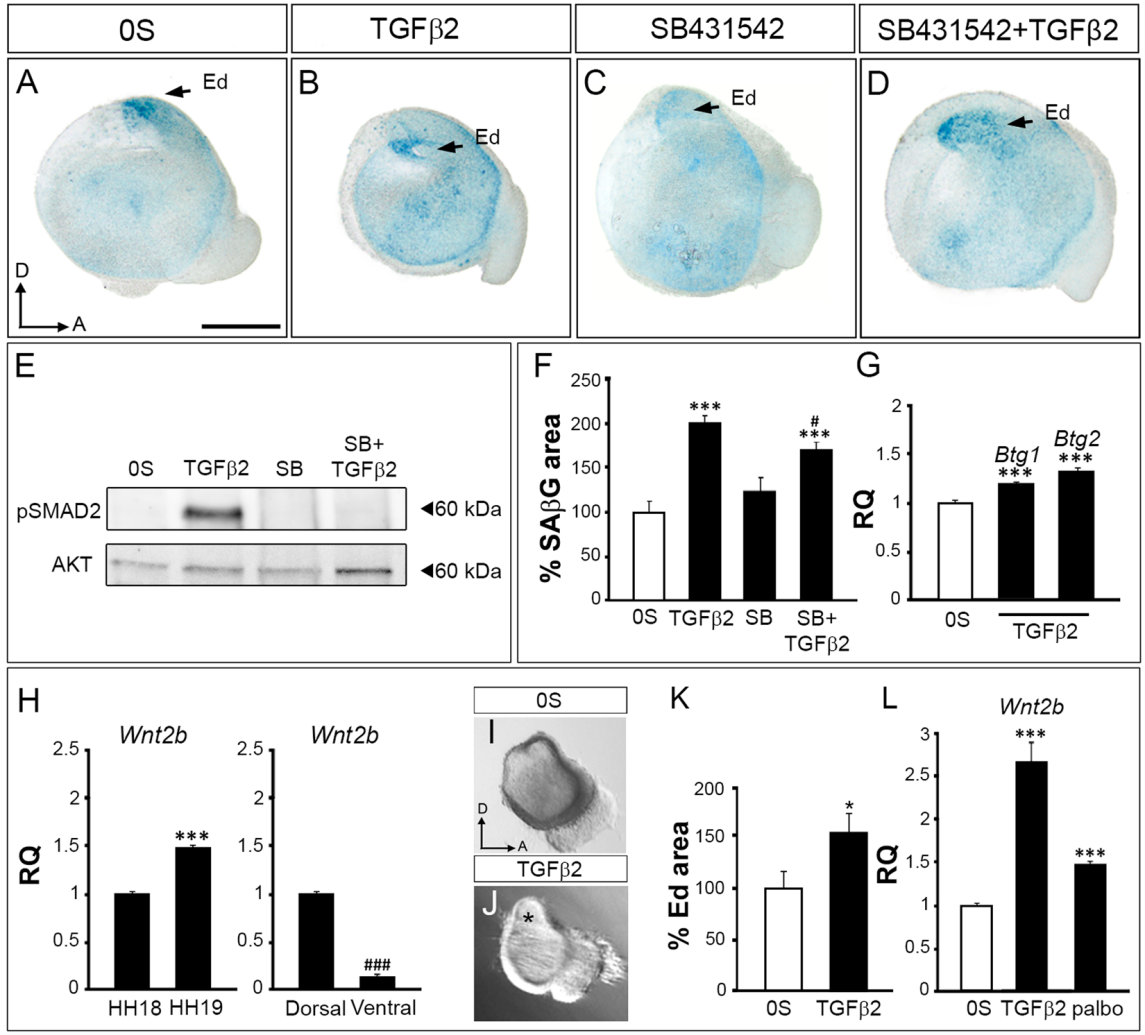
TGFβ pathway induction of senescence in the otocyst. (**A**–**D**) SAβG staining in isolated otic vesicles from stage HH18 embryos cultured *ex vivo* for 20 h in free serum medium (0S), in the presence of TGFβ2 (10 ng/ml), SB (SB431542; 10 µM) or a combination of both. (**E**) Levels of pSMAD2 were measured by western blotting of explanted otic vesicle lysates (two independent experiments). (**F**) SAβG staining quantification is shown as mean SAβG area ± SEM with respect to the total area, normalized to the 0S condition (white bar). ***p < 0.001 vs 0S; ^#^p < 0.05 vs SB. (**G**) *Btg1* and *Btg2* mRNA expression levels were measured by RT-qPCR in cultured otic vesicles. Statistical significance was determined with the Student’s t-test: ***P < 0.005, versus 0S. (**H**) *Wnt2b* mRNA expression levels were measured by RT-qPCR in non-cultured HH18 and HH19 otic vesicles and in dissected dorsal and ventral regions from HH19 otic vesicles. Statistical significance was determined with the Student’s t-test: ***P < 0.005, versus HH18; ^###^P < 0.005, versus HH19 dorsal regions. (**I**,**J**) Light microscopy images from TGFβ2-treated otic vesicles showing the relative increment of the endolymphatic duct area (**J**, asterisk, quantification in **K**). Quantification for areas were measured and are shown as the mean ± SEM and the statistical significance was determined by Student’s t-test: *P < 0.05, versus control condition (0S, white bars). (**L**) *Wnt2b* mRNA levels were measured by RT-qPCR in cultured otic vesicles in 0S medium, in presence of TGFβ2 (10 ng/ml) or palbociclib (1 µM). Statistical significance was determined with the Student’s t-test: ***P < 0.005, versus 0S. Gene expression was calculated as 2^−ΔΔCt^ and normalized to the levels of 0S (white bars). The results are expressed as the mean ± SEM (three independent experiments performed in triplicate). Ed, endolymphatic duct. Orientation: A, anterior; D, dorsal. Scale bar: 150 μm.

Otic vesicles (HH18) were isolated and cultured with or without exogenous TGFβ2 (Fig. 4). TGFβ2 significantly increased SAβG by 2-fold (Fig. 4A,B, quantification in F) and increased *Btg1* and *Btg2* expression levels (1.18-fold and 1.33-fold respectively; Fig. 4G). To further confirm the role of TGFβ2 in the regulation of otic senescence, otic vesicle explants were treated with the inhibitor of TGFβ signalling SB431542^34^. TGFβ2-induced SMAD2 phosphorylation was reduced by co-treatment with SB431542 (Fig. 4E), and SB431542 also reduced TGFβ2-induced SAβG staining (Fig. 4C,D, quantification in F). In contrast, SAβG staining in the control condition (0S) was not modified by SB431542.

To explore if TGFβ2 modulates endolymphatic duct morphogenesis we analysed the morphology of the otocyst as well as the expression levels of *Wnt2b*, which is specifically expressed in the mouse endolymphatic duct^35^. We confirmed that *Wnt2b* is expressed in the chicken endolymphatic duct by showing its upregulation (1.47-fold) in HH19 otic vesicle explants. Concordantly, *Wnt2b* expression was higher (7.3-fold) in dissected dorsal regions of HH19 otic explants compared to ventral regions (Fig. 4H). In parallel, the ventral-expressed *Neurog1* was downregulated in HH19 (0.52-fold) consistently with its role in early neurogenesis^3^ and the expression levels were upregulated (2-fold) in the ventral region compared to the dorsal region (Supplementary Information Fig. S3). The association between cellular senescence and endolymphatic duct development was further confirmed by the differentiation of the endolymphatic duct observed following TGFβ2 treatment (Fig. 4I,J, asterisk, quantification in K). This was consistent with the significant increase of *Wnt2b* found in otic vesicles treated with either TGFβ2 (3-fold) or palbociclib (1.47-fold; Fig. 4L). Therefore, we conclude that TGFβ2 plays an inductive role in endolymphatic duct morphogenesis by promoting cellular senescence.

### PI3K/AKT and RAF-MEK-ERK pathways modulate senescence in the otic vesicle

**Figure 5:**
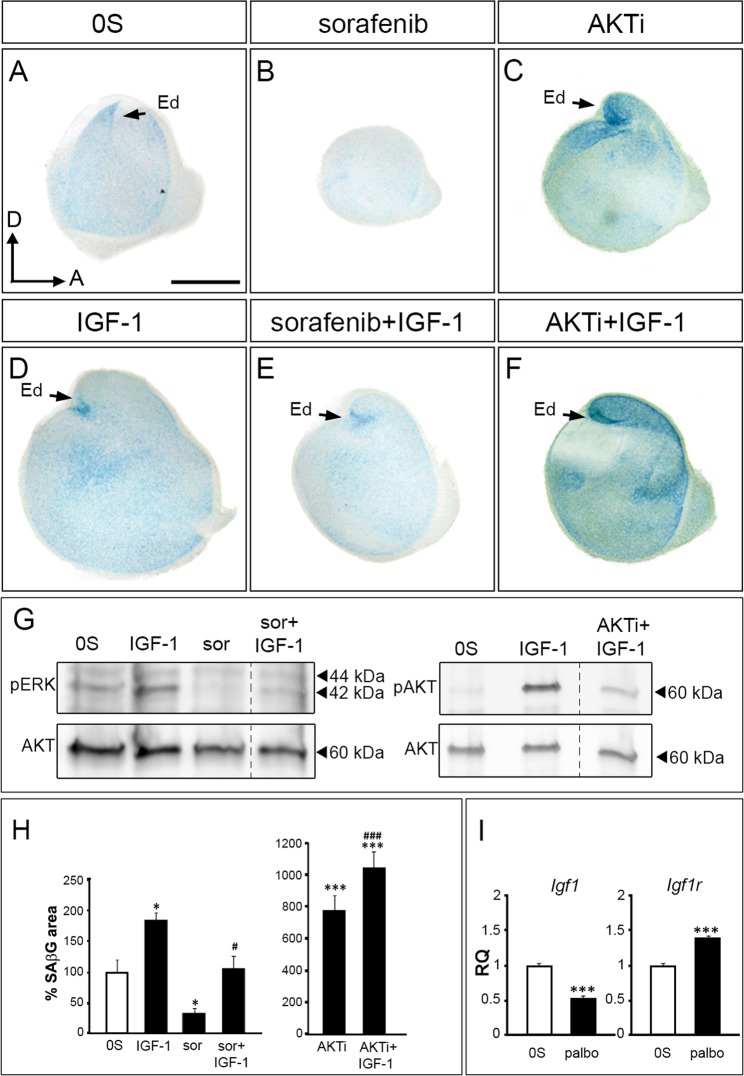
IGF-1 modulates developmental senescence of chicken inner ear. (**A**–**F**) SAβG staining in isolated otic vesicles from stage HH18 embryos cultured *ex vivo* for 20 h in free serum medium (0S), in the presence of IGF-1 (10 nM), Sor (2.5 µM), AKTi (50 μM) or a combination. (**G**) Phosphorylation state of IGF-1 targets in HH18 otic vesicles explanted and incubated with the indicated treatments. pERK, pAKT and AKT were measured by western blotting of at least n = 15 otic vesicle lysates in two independent experiments. To improve the clarity of the presentation lanes have been grouped. Full-length blots are presented in Supplementary Figure S3. (**H**) SAβG staining quantification by FIJI software. Data are shown as mean SAβG area ± SEM with respect to the otic vesicle area, normalized to the 0S condition. *p < 0.05, ***p < 0.001 vs 0S; ^#^p < 0.05, ^###^p < 0.001 vs. indicated inhibitor. (**I)** *Igf1 and Igf1r* mRNA levels from explanted HH18 otic vesicles in the absence (0S, white bars) or presence of palbociclib (1 µM). Gene expression was calculated as 2^−ΔΔCt^ and normalized to the levels of the control condition (0S). The results are expressed as the means ± SEM from three independent experiments performed in triplicate. Statistical significance was determined with the Student’s t-test: ***P < 0.005, versus 0S. Ed, endolymphatic duct. Orientation: A, anterior; D, dorsal. Scale bar: 150 μm.

Based on previous findings from our lab^17,19^, IGF-1 was used as an activator of otic PI3K/AKT and RAF-MEK-ERK pathways. Figure 5 shows a significant decrease (70%) of SAβG staining in the presence of the RAF kinase inhibitor sorafenib (Fig. 5A,B, quantification in H), whereas an increase was observed when the activity of AKT was inhibited (7.7-fold; Fig. 5A,C, quantification in H). Treatment with AKTi or sorafenib inhibited the phosphorylation of AKT and ERK, respectively (Fig. 5G). To further understand the interplay between the extrinsic factors that regulate these pathways, cellular senescence was studied following IGF-1-stimulation (Fig. 5D–F). SAβG staining was increased by IGF-1 (1.9-fold), which was able to partially overcome the effects of sorafenib but not those of AKTi that were even potentiated (Fig. 5E,F quantification in 5 H). In contrast, palbociclib-induced senescence showed reduced *Igf1* transcript levels (0.52-fold; Fig. 5I) but increased *Igf1r* expression (1.37-fold). These results are in agreement with the arrested proliferative status of senescent cells (Figs 1J and 2Q,R) and the reported proliferative role of IGF-1^36^. Upregulation of IGF1R transcription is a typical compensatory cell response to reduced IGF-1 signalling^37,38^.

## Discussion

Here we show that senescence plays a role in the early development of the endolymphatic duct, site of intense programmed cell death and reduced proliferation. Elimination of senescent cells and induction of senescence in the otic vesicles showed opposing morphogenetic defects. Our results show that senescence is induced by TGFβ2 in the otic vesicle, where TGFβ pathway components are expressed at these early embryonic stages. In addition, IGF-1 targeted kinases AKT and RAF modulated SAβG staining in the otic vesicle.

Developmental senescence in the otic vesicle is confirmed by specific SAβG staining, arrested proliferation and senescence related gene transcript upregulation. *Btg1* and *Btg2* appeared to be particularly relevant as senescence read-outs in chicken. Both genes are differentially regulated in limb samples of species that eliminate the interdigital tissue by senescence (chicken) compared to those that doesn´t (duck). *Btg1* and *Btg2* are both upregulated during interdigital tissue regression (7.5–8d) in chick limbs, while in the duck limbs *Btg1* is upregulated but *Btg2* is downregulated^14^. Our results show that *Btg1* and *Btg2* expression is modulated during otic development, being both upregulated at HH19 at the time of endolymphatic duct formation. *Btg1* and *Btg2* roles in senescence was further confirmed by their induction with palbociclib and TGFβ2.

Senescence is very intense at the edges of the pores of structures that arise from ectoderm invaginations which eventually close up, such as the otic pore and the lens vesicle pore. Apoptosis is also present at pore edges during the otic cup stage, which associates with the folding and subsequent closure and separation of the otic vesicle from the ectoderm^4^. These results suggest a connection between senescence and apoptosis in promoting the closure of the otic cup to form the otocyst. The concurrence of senescence and apoptosis during development has been recently demonstrated in the vertebrate limb, particularly in the AER and the elimination of the interdigital tissue^13,14^. Our work also shows that senescence staining and TUNEL labelling coincides in specific areas of the endolymphatic duct also showing signs of cell cycle arrest. Therefore, our data support the presence of developmental senescence concomitant with reported areas of apoptosis in the otocyst^4^.

Research on senescence during development published to date shows that underlying molecular mechanisms and function vary depending on the structure. Developmental senescence in the interdigital webs of the developing limb is followed by apoptosis and subsequently removed by macrophage-mediated phagocytosis^14^. Mesonephros elimination is due to senescent cells that are also subsequently removed by macrophage-mediated phagocytosis^12^. This cell-clearing function might be conserved in all vertebrates studied from teleost fish to mice^39^. Thus, senescence might be a conserved mechanism that facilitates the elimination of the excess of cells generated during development.

Work from us and others show developmental senescence at regions that undergo morphological changes and generate three-dimensional structures such as the dorsal region of the pharyngeal slits, the edges of the optic lens vesicle pores, the pineal gland and the otic vesicle^13^. Moreover, senescence has been reported to be a remodelling mechanism in *Xenopus laevis* cement gland^40^. The morphological changes that the HH18 otocyst undergoes include the protrusion of the endolymphatic duct. Elimination of senescent cells abolished endolymphatic duct morphogenesis. It would therefore be reasonable to propose here that senescence facilitates the acquisition of the three-dimensional otic vesicle shape, first by blocking proliferation and second by promoting the slow removal of cells.

Our results also suggest a role for developmental senescence in otic dorso-ventral patterning. The phenotype of otic vesicles treated with palbociclib showed a dorsalized otocyst with increased SAβG staining in the ventral region. This result might be explained by the ectopic secretion in the ventral region of SASP component(s) that could promote dorsalization. In fact, the palbociclib-induced phenotype is related to that of the effect of BMP4 overexpression which also induces thinning and ventral expansion of the dorsal epithelium^41^.

Later in development, senescence facilitates the maturation of the endolymphatic sac by favouring the presence of pendrin-positive cells^12^, which are essential for the maintenance of the endolymph in the adult inner ear^42^. Thus, senescence during the developing inner ear plays different roles sequentially, first sculpting the endolymphatic duct in the otocyst and later defining endolymphatic sac cell populations.

TGFβ2 plays an inductive role in programmed senescence during mouse embryonic development^12^. Our data show that TGFβ2 also promotes senescence during inner ear development. *Tgfβ2* is expressed in the otic epithelium with the highest levels observed in the dorsal part of the otocyst, including the endolymphatic duct anlagen. Its target pSMAD2 was also present in these areas. In our hands, TGFβ2-treatment potentiates endolymphatic duct formation through senescence. These results are particularly interesting because inhibition of BMP-ligands reduces differentiation of dorsal otic areas without affecting endolymphatic duct formation^41^. Therefore, our work proposes a specific role for TGFβ2 on the endolymphatic duct morphogenesis by means of inducing senescence. The specific expression of TGFβ2 in the endolymphatic duct at HH18 and HH20 reinforce the proposed actions for TGFβ2 in the formation of the endolymphatic duct. Further supporting this view is the fact that TGFβ2 deficient mice show morphological defects and alterations in the homeostasis of endolymph^43^.

Surprisingly, in the absence of an exogenous source of TGFβ2, blocking endogenous TGFβ signalling with SB431542 does not suffice to decrease otic senescence. This highlights the fact that the inhibitor may not be completely inhibiting its target, TGFβR1. The small reduction in the magnitude of the phenotype upon addition of both SB431542 and TGFβ2 might explain why we do not observe a significant reduction in the senescence with the inhibitor alone as compared to the control condition (0S).

However, we cannot exclude that other senescence-modulating players could be playing a role in compensating the absence of complete TGFβ signaling. One strong candidate might be IGF-1.

Our results showed that palbociclib inhibited cell proliferation and reduced *Igf1* expression. IGF-1 is a crucial survival and proliferation factor for otic precursors in a variety of species from chicken to mammals^16,44^. IGF-1 actions in the developing inner ear are mainly mediated by the RAF-MEK-ERK cascade and the PI3K/AKT pathway^17,19^. Inhibition of PI3K/AKT increased senescence in the otocyst, suggesting that this pathway is repressing senescence as reported during mouse development^12^. On the contrary, the inhibition of RAF kinases by sorafenib reduces senescence, suggesting that this pathway promotes both proliferation and senescence. Accordingly, Storer *et al*., 2013 showed the non-autonomous involvement of the RAF-MEK-ERK pathway in the induction of developmental senescence in the mouse AER. These data suggest that senescence could be one of the mechanisms activated secondarily to proliferation to control the extent of cell division needed to sculpt the otocyst. B-RAF expression shows a highly specific pattern in the chicken developing inner ear that is very intense in the endolymphatic duct^19^, thus our data also suggest that the degree of activation of RAF kinases might be the trigger that switches cell fate from proliferation to senescence. Accordingly, it has been reported that increased B-RAF activity may correlate with cell cycle arrest and the expression of typical cell senescence markers^45^.

IGF-1 is a survival factor whose proliferative actions have been well-documented^36^. Our data show that IGF-1 induced proliferation and survival but also an increase in the number of cell cycle-arrested senescent cells. Therefore, our data suggest that as a consequence of IGF-1 strong induction of proliferation there is an immediate induction of senescence to sculpt the otocyst in those areas where a more active remodelling is needed, as in the endolymphatic duct. Indeed, IGF-1 treatment during 24 h turns a rounded otic vesicle into a pear-shaped structure with the primordium of the endolymphatic duct evidenced at the most dorsal region^46^. The fact that IGF-1, IGF1R and B-RAF are expressed at the endolymphatic duct during chicken otic development^16,19^, is consistent with this hypothesis.

In summary, our work shows that cellular senescence occurs in the developing inner ear. TGFβ2 is a potent inductor of senescence that may be instructing endolymphatic duct morphogenesis. We also show here that cell proliferation induced by IGF-1 via activation of RAF kinases modulates developmental senescence and, in turn, morphogenetic events.

## Supplementary information

Supplementary Information

## Data Availability

The datasets generated during and/or analysed during the current study are available from the corresponding author on reasonable request.
