# Supplementary material for: TGFβ2-induced senescence during early inner ear development
Source: Sci Rep. 2019 Apr 11;9:5912. doi: 10.1038/s41598-019-42040-0 (PMC6459823; doi:10.1038/s41598-019-42040-0)
Supplement: Supplementary file 1 — Supplementary Information [file 41598_2019_42040_MOESM1_ESM.pdf]

TGFβ2 -induced senescence during early inner ear development

Alejandro Gibaja[1*], María R. Aburto[1*], Sara Pulido[1,2], Manuel Collado[3], Juan M. Hurlé[4], Isabel Varela-Nieto[1,4,5#] and Marta Magariños[1,2,6#]

[1] "Alberto Sols" Biomedical Research Institute (CSIC-UAM), Madrid, Spain.
[2] CIBERER, Institute of Health Carlos III, Madrid, Spain.
[3] Instituto de Investigación Sanitaria de Santiago de Compostela, Santiago de Compostela, Spain.
[4] Departamento de Anatomía y Biología Celular and IDIVAL, Universidad de Cantabria. Santander, Spain.
[5] IdiPAZ, La Paz Hospital Institute for Health Research, Madrid, Spain.
[6] Department of Biology, Autonomous University of Madrid, Madrid, Spain.

* Equal contribution
# Equal senior contribution and corresponding authors

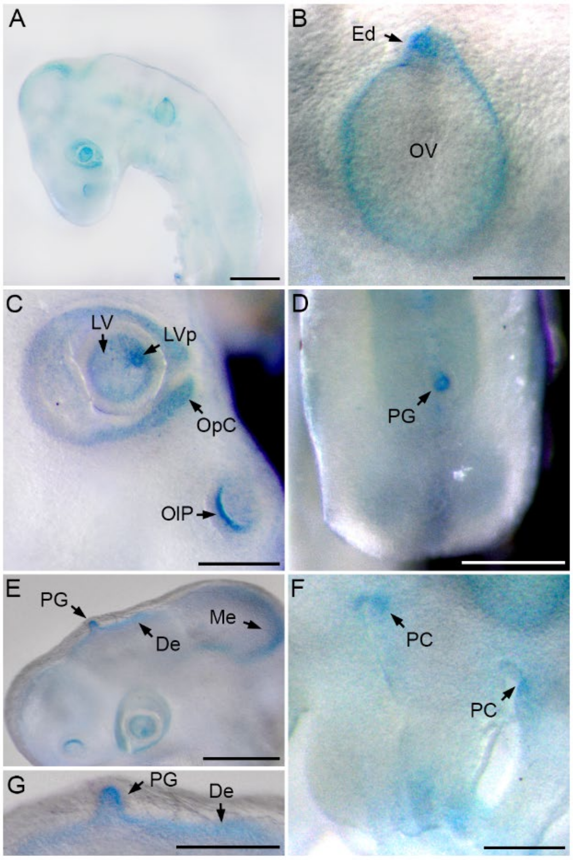

Supplementary Information Figure S1. **Senescence during the early development of the chicken embryo.** Senescence was visualised in specific regions of chicken embryos by SAβG staining. **A**) Overview of SAβG staining in the HH20 embryo; **B**) Close up the otic vesicle from an HH20 embryo showing senescence in the otic epithelium particularly intense at the endolymphatic duct; **C**) SAβG staining in the optic cup, in the lens vesicle and in the olfactory placode at HH17; **D**) SAβG staining in the pineal gland of an HH17 stage embryo; **E**) Overview of the head of an HH17 embryo showing senescence in the diencephalon, midbrain, pineal gland; **F**) SAβG staining in the dorsal region of the pharyngeal clefts; **G**) Higher magnification of the epiphysis and diencephalon in E showing SAβG staining. Abbreviations: De, diencephalon; Ed, endolymphatic duct; LV, lens vesicle; LVp, lens vesicle pore; Me, midbrain; OlP, olfactory placode; OpC, optic cup; PC, pharyngeal cleft; PG, pineal gland; OV, otic vesicle. Scale bars: 500

μm (A, E); 250 μm (C, D, F, G); 125 μm (B). Representative microphotographs are shown of at least n=2-3 stained embryos.

TGFβ2 -induced senescence during early inner ear development

Alejandro Gibaja[1*], María R. Aburto[1*], Sara Pulido[1,2], Manuel Collado[3], Juan M. Hurlé[4], Isabel Varela-Nieto[1,4,5#] and Marta Magariños[1,2,6#]

[1] "Alberto Sols" Biomedical Research Institute (CSIC-UAM), Madrid, Spain.
[2] CIBERER, Institute of Health Carlos III, Madrid, Spain.
[3] Instituto de Investigación Sanitaria de Santiago de Compostela, Santiago de Compostela, Spain.
[4] Departamento de Anatomía y Biología Celular and IDIVAL, Universidad de Cantabria. Santander, Spain.
[5] IdiPAZ, La Paz Hospital Institute for Health Research, Madrid, Spain.
[6] Department of Biology, Autonomous University of Madrid, Madrid, Spain.

* Equal contribution
# Equal senior contribution and corresponding authors

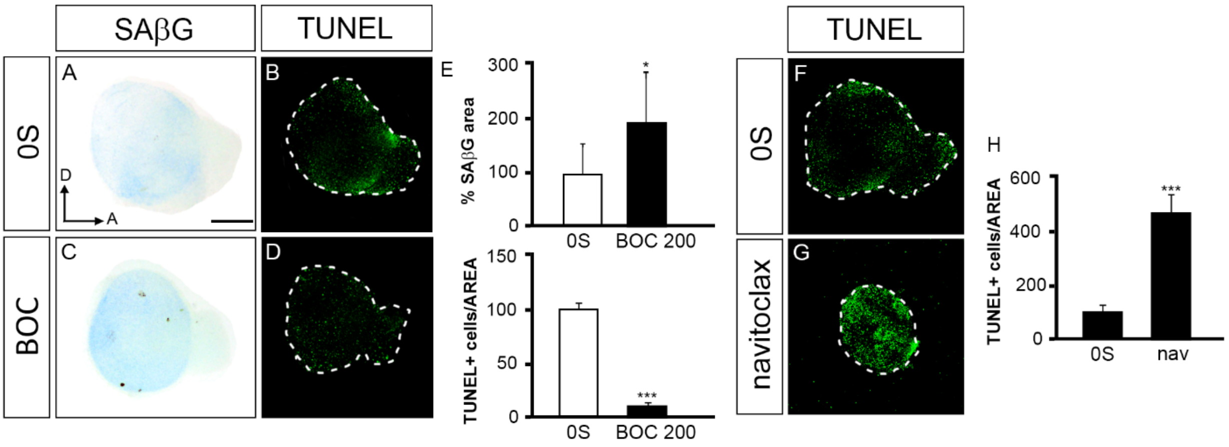

Supplementary Information Figure S2. Senescence and apoptosis in explanted chicken otic vesicles. Otic vesicles were isolated from HH18 chicken embryos and incubated ex vivo for 20 h in serum-free culture medium without additives (0S, A-B,F), in the presence of the pan-caspase inhibitor BOC (200 μM; C-D) or in the presence of the senolytic navitoclax (1 μM; G). SAβG staining was increased by 1.9-fold in BOC-treated otic vesicles (A,C, quantification in E), whilst apoptotic cell death visualized by TUNEL staining (green) was reduced by 0.9-fold (B,D, quantification in E). Elimination of senescent cells by navitoclax reduced SAβG staining (Figure 2 A,E, quantification in M) and increased apoptosis (F,G, quantification in H). SAβG staining is represented with respect to the total otic vesicle area. TUNEL-positive cells were measured from compiled confocal microscopy projections and normalized to the 0S condition. At least n=4 otic vesicles per condition were studied. Data are shown as mean ± SEM.*$p<0.05$, ***$p<0.001$ vs 0S. of otic vesicles. Orientation: A, anterior; D, dorsal. Scale bars, 150 μm.

TGFβ2 -induced senescence during early inner ear development

Alejandro Gibaja[1*], María R. Aburto[1*], Sara Pulido[1,2], Manuel Collado[3], Juan M. Hurlé[4], Isabel Varela-Nieto[1,4,5#] and Marta Magariños[1,2,6#]

[1] "Alberto Sols" Biomedical Research Institute (CSIC-UAM), Madrid, Spain.
[2] CIBERER, Institute of Health Carlos III, Madrid, Spain.
[3] Instituto de Investigación Sanitaria de Santiago de Compostela, Santiago de Compostela, Spain.
[4] Departamento de Anatomía y Biología Celular and IDIVAL, Universidad de Cantabria. Santander, Spain.
[5] IdiPAZ, La Paz Hospital Institute for Health Research, Madrid, Spain.
[6] Department of Biology, Autonomous University of Madrid, Madrid, Spain.

* Equal contribution
# Equal senior contribution and corresponding authors

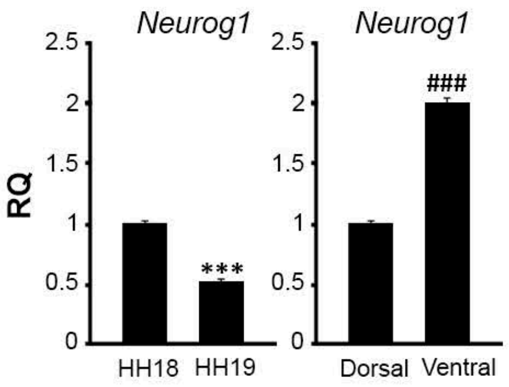

Supplementary Information Figure S3. *Neurog1* as a biomarker of early ventral region. *Neurog1* mRNA expression levels were measured by RT-qPCR in non-cultured HH18 and HH19 otic vesicles and in dissected dorsal and ventral regions HH19 otic vesicles. Statistical significance was determined with the Student's t-test: ***P<0.005, versus HH18; ###P<0.005, versus dorsal region.

TGFβ2 -induced senescence during early inner ear development

Alejandro Gibaja[1*], María R. Aburto[1*], Sara Pulido[1,2], Manuel Collado[3], Juan M. Hurlé[4], Isabel Varela-Nieto[1,4,5#] and Marta Magariños[1,2,6#]

[1] "Alberto Sols" Biomedical Research Institute (CSIC-UAM), Madrid, Spain.
[2] CIBERER, Institute of Health Carlos III, Madrid, Spain.
[3] Instituto de Investigación Sanitaria de Santiago de Compostela, Santiago de Compostela, Spain.
[4] Departamento de Anatomía y Biología Celular and IDIVAL, Universidad de Cantabria. Santander, Spain.
[5] IdiPAZ, La Paz Hospital Institute for Health Research, Madrid, Spain.
[6] Department of Biology, Autonomous University of Madrid, Madrid, Spain.

* Equal contribution
# Equal senior contribution and corresponding authors

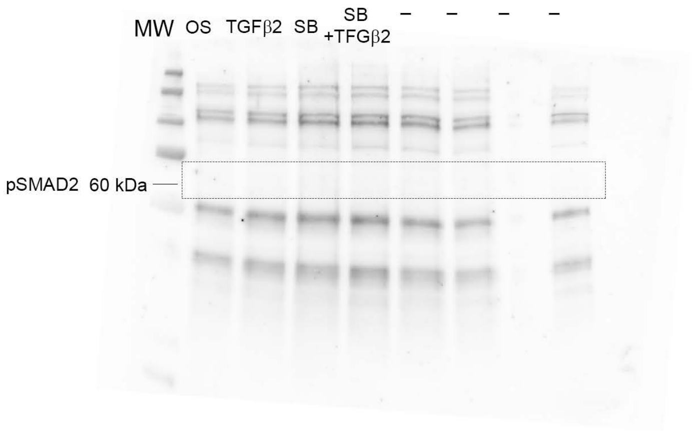

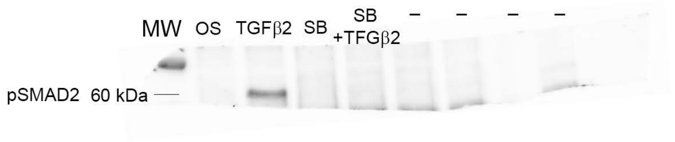

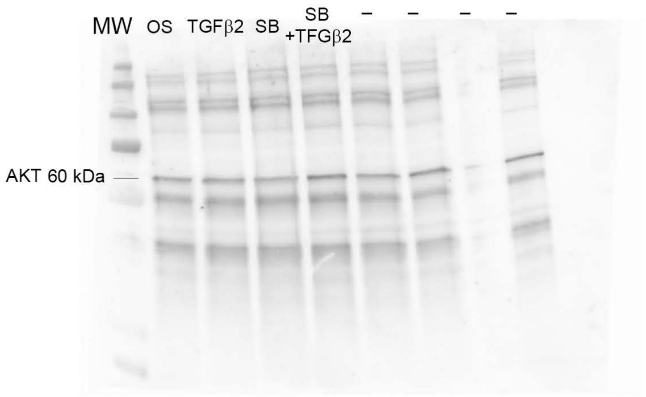

Supplementary Information S4. Full length blots. To improve the clarity of the information, we grouped cropped blots in Figure 4 western blottings in which we excluded conditions that were not objective of these manuscript (shown in the images as "-").

TGFβ2 -induced senescence during early inner ear development

Alejandro Gibaja[1*], María R. Aburto[1*], Sara Pulido[1,2], Manuel Collado[3], Juan M. Hurlé[4], Isabel Varela-Nieto[1,4,5#] and Marta Magariños[1,2,6#]

[1] "Alberto Sols" Biomedical Research Institute (CSIC-UAM), Madrid, Spain.
[2] CIBERER, Institute of Health Carlos III, Madrid, Spain.
[3] Instituto de Investigación Sanitaria de Santiago de Compostela, Santiago de Compostela, Spain.
[4] Departamento de Anatomía y Biología Celular and IDIVAL, Universidad de Cantabria. Santander, Spain.
[5] IdiPAZ, La Paz Hospital Institute for Health Research, Madrid, Spain.
[6] Department of Biology, Autonomous University of Madrid, Madrid, Spain.

* Equal contribution
# Equal senior contribution and corresponding authors

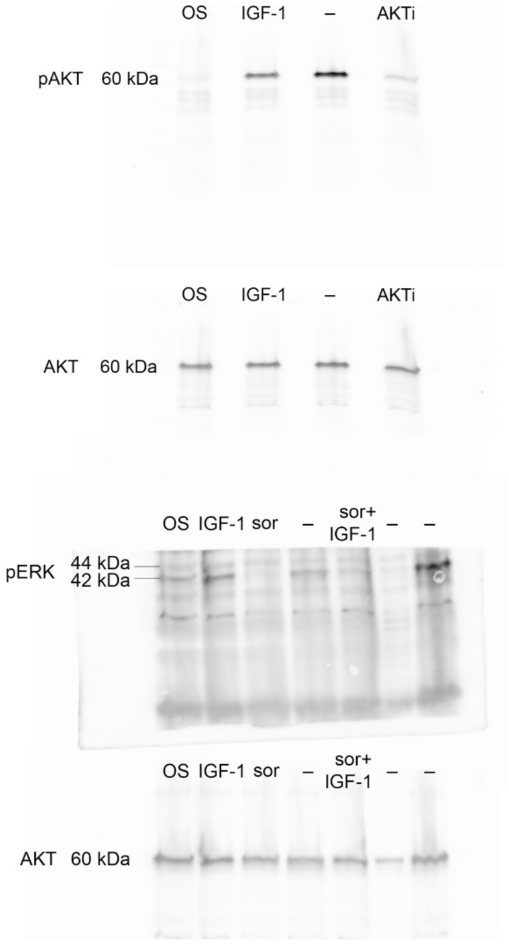

Supplementary Information S5 Full length blots. To improve the clarity of the information, we grouped cropped blots in Figure 5western blottings in which we excluded conditions that were not objective of these manuscript (shown in the images as "-"). The antibodies used (Supplementary Information Table S1) have been previously characterized upon the same conditions in otic vesicles cultures (references 17 and 19).

TGFβ2-induced senescence during early inner ear development

Alejandro Gibaja[1*], María R. Aburto[1*], Sara Pulido[1,2], Manuel Collado[3], Juan M. Hurlé[4], Isabel Varela-Nieto[1,4,5#] and Marta Magariños[1,2,6#]

[1] "Alberto Sols" Biomedical Research Institute (CSIC-UAM), Madrid, Spain.
[2] CIBERER, Institute of Health Carlos III, Madrid, Spain.
[3] Instituto de Investigación Sanitaria de Santiago de Compostela, Santiago de Compostela, Spain.
[4] Departamento de Anatomía y Biología Celular and IDIVAL, Universidad de Cantabria. Santander, Spain.
[5] IdiPAZ, La Paz Hospital Institute for Health Research, Madrid, Spain.
[6] Department of Biology, Autonomous University of Madrid, Madrid, Spain.

* Equal contribution
# Equal senior contribution and corresponding authors

Supplementary Information Table S1. Antibodies used

Antibody	*Type[1]*	*Source/Cat #*	*Technique*
**Anti-Akt1/2**	GP	Santa Cruz/sc-1619	1:1000 (WB)
**Anti-pAkt^{Ser473}**	RbP	Cell Signaling/9271	1:1000 (WB)
**Anti-pSMAD2**	RbP	Cell Signaling/3101	1:1000 (WB), 1:200 (IHF)
**Anti-ERK MAPK**	RbP	Cell Signaling/9102	1:1000 (WB)
**Anti-pERK MAPK**	RbP	Cell Signaling/ 9101	1:1000 (WB)
**Anti-phistone 3**	Rbp	Upstate/06-570	1:200 (IHF)

Summary of antibodies used for immunohistofluorescence (IHF) and western blotting (WB). [1]Antibody type: RbP, rabbit polyclonal; GP, goat polyclonal.
